# Exploring Locality Sensitive Hashing as a Blocking Method for Large-Scale Administrative Datasets

**DOI:** 10.23889/ijpds.v9i5.2863

**Published:** 2024-09-18

**Authors:** Josie Plachta, Rachel Shipsey, Leah Quinn

**Affiliations:** 1 Office for National Statistics

## Objectives

Linking large-scale datasets is challenging due to the computational power required. This research explores using Locality-Sensitive-Hashing (LSH) as a novel blocking method to facilitate linking large administrative data. LSH uses an efficient technique of hashing data while preserving similarity, reducing the search space and processing power required to find links.

## Approach

Samples were taken from a gold-standard dataset and blocked using LSH. Various parameters were tested to establish LSH’s optimal performance at retrieving linked pairs and reducing the search space size.

## Results

Testing the method on small datasets gave promising results, with the LSH method creating ~9,000 candidate pairs. This is a improvement over our traditional blocking method or cartesian product, which created ~70,000 and 23.4 million candidate pairs, respectively. We have therefore shown that LSH can significantly reduce the search-space size. A further advantage of the method is its capability in handling alternative variables such as those that may be present in longitudinal or composite data, without needing to manually anticipate the different combinations of variables which may occur. Variable formats to simulate agreement weighting were also considered, with encouraging results.

## Conclusion and Implications

Our research shows that LSH can be used to drastically reduce the search space when blocking for data linkage This suggests developing LSH as a blocking method could result in more effective blocking, quicker linkage, and no loss of quality compared to traditional methods. However, further research on increasing the method’s scale is necessary.

